# Diabetes Risk Reduction Diet and Endometrial Cancer Risk

**DOI:** 10.3390/nu13082630

**Published:** 2021-07-30

**Authors:** Giovanna Esposito, Francesca Bravi, Diego Serraino, Fabio Parazzini, Anna Crispo, Livia S. A. Augustin, Eva Negri, Carlo La Vecchia, Federica Turati

**Affiliations:** 1 Department of Clinical Sciences and Community Health, University of Milan, 20122 Milano, Italy; giovanna.esposito@unimi.it (G.E.); francesca.bravi@unimi.it (F.B.); fabio.parazzini@unimi.it (F.P.); eva.negri@unimi.it (E.N.); carlo.lavecchia@unimi.it (C.L.V.); 2 Unit of Cancer Epidemiology, CRO Aviano National Cancer Institute, IRCCS, 33080 Aviano, Italy; serrainod@cro.it; 3 Department of Obstetrics, Gynecology and Neonatology—Fondazione IRCCS Ca’ Granda Ospedale Maggiore Policlinico, 20122 Milano, Italy; 4 Epidemiology and Biostatistics Unit, Istituto Nazionale dei Tumori—IRCCS “Fondazione G. Pascale”, 80131 Napoli, Italy; a.crispo@istitutotumori.na.it (A.C.); l.augustin@istitutotumori.na.it (L.S.A.A.); 5 Department of Humanities, Pegaso Online University, 80143 Napoli, Italy; 6 Unit of Medical Statistics and Biometry, Fondazione IRCCS Istituto Nazionale Dei Tumori Di Milano, 20122 Milano, Italy

**Keywords:** endometrial cancer, case–control study, diabetes, diabetes risk reduction diet, dietary patterns

## Abstract

Diabetes increases endometrial cancer risk. We investigated the role of a diabetes risk reduction diet (DRRD) on the risk of endometrial cancer using data from a multicentric, Italian hospital-based case–control study (1992–2006) enrolling 454 histologically confirmed cases of endometrial cancer and 908 controls matched by age and center. We derived a DRRD score assigning higher scores for higher intakes of cereal fiber, fruit, coffee, polyunsaturated:saturated fatty acid ratio, and nuts and for lower glycemic load and lower intakes of red/processed meat and sugar-sweetened beverages/fruit juices. The odds ratios (OR) of endometrial cancer according to the DRRD score were derived by multiple conditional logistic regression models. The OR for high (DRRD score >24, i.e., third tertile) versus medium–low adherence to the DRRD was 0.73 (95% confidence interval, CI, 0.55–0.97). Similar results were observed after the exclusion of diabetic women (OR 0.75; 95% CI, 0.56–1.00) and allowance for total vegetable consumption (OR 0.80; 95% CI, 0.60–1.07). Inverse associations were observed in most of the analyzed subgroups. The OR for high DRRD combined with high vegetable consumption was 0.45 (95% CI, 0.28–0.73). Our results suggest that diets able to reduce diabetes risk may also reduce endometrial cancer risk. High vegetable consumption combined with high adherence to the DRRD may provide additional benefit in endometrial cancer prevention.

## 1. Introduction

Endometrial cancer arises predominantly in post-menopausal women [1]. Risk factors for the disease include obesity [2,3,4], physical inactivity [3,5], nulliparity, early age at menarche [6,7], estrogenic hormone replacement therapy (HRT) [8], diabetes, hyperinsulinemia, and insulin resistance [9,10]. Inflammation and oxidative stress may also increase the risk [11]. Conversely, combined oral contraceptives (OC) [12] and cigarette smoking [13] are inversely associated with endometrial cancer risk.

Several studies found an increased risk of endometrial cancer in women with diabetes [4,14,15,16,17], by approximately 70% [16]. Obesity, the metabolic syndrome [18], and insulin resistance are well-recognized correlates of endometrial cancer. They represent a hallmark among women affected by diabetes mellitus, though the association with diabetes is only partly accounted for by higher body mass index (BMI) [10].

Diet may impact endometrial cancer independently from obesity, but the exact underlying mechanisms remain poorly understood [19]. Coffee intake was consistently inversely associated with the risk of endometrial cancer [20,21,22]. A direct association between a high glycemic load (GL) diet and the risk of endometrial cancer was reported by several studies [23,24], but the evidence is not fully consistent [25,26,27]. Evidence is suggestive but inconclusive for a direct association with the intake of red and processed meat [28,29,30,31,32] and for inverse associations with the intakes of cereal fiber [33,34,35] and fruit [30,32,36].

Diet is known to influence insulin resistance and hyperinsulinemia. Along this line, a dietary pattern for diabetes mellitus prevention (the diabetes risk reduction diet (DRRD)) was recently proposed [37]. The original version of the DRRD was characterized by high intakes of cereal fiber, coffee and nuts, high polyunsaturated:saturated fats ratio, low glycemic index (GI), and low intakes of red and processed meat, sugar-sweetened beverages, and trans fats. Higher adherence to the original DRRD was found to decrease the risk of hepatocellular carcinoma [38]. More recently, a new version of the DRRD was developed, where fruit was added as a favorable component and fruit juices were included in the sugar-sweetened beverage unfavorable component. The authors observed an inverse association with the risk of breast cancer [39]. Similarly, high adherence to the DRRD was associated with a reduced risk of breast cancer in an Italian case–control study [40]. In another study, the DRRD was inversely related to pancreatic cancer [41]. The effect of this dietary approach on the risk of endometrial cancer has not yet been evaluated.

In the present investigation, we assessed the association between adherence to the DRRD and the risk of endometrial cancer using data derived from a multicentric Italian study.

## 2. Materials and Methods

We retrieved data from a multicentric case–control study on endometrial cancer carried out between 1992 and 2006 in three Italian areas, i.e., the metropolitan area of Milan, the provinces of Udine and Pordenone in northern Italy, and the urban Naples area in southern Italy [30,42]. The study included 454 incident cases (median age 60 years, range 18–79) and 908 controls (median age 61 years, range 19–79) frequency matched by quinquennia of age and study center with a 2:1 ratio.

Cases were women with a diagnosis of endometrial cancer, histologically confirmed, according to the International Classification of Diseases (ICD-9-CM, code 182.0), hospitalized in the major university and general hospitals of the study areas. Women diagnosed with an endometrial cancer up to a year earlier and with no previous cancer at any site were eligible. Controls, enrolled in the same network of hospitals as cases, were women admitted for a wide spectrum of acute and non-neoplastic illnesses: traumas (36%); other orthopedic disorders (32%); acute surgical conditions (9%); and miscellaneous illnesses including eye, nose, ear, or skin disorders (23%). Among controls, we excluded women with a previous history of hysterectomy or those hospitalized for hormone-related or gynecological conditions, or any clinical condition leading to long-term dietary changes. Over 95% of women approached agreed to take part in the study.

Centrally trained personnel interviewed cases and controls during their hospital stay using a standard structured questionnaire, including sociodemographic and anthropometric characteristics, selected lifestyle behaviors (i.e., tobacco smoking, alcohol consumption, and physical activity), personal clinical information, family (first-degree relatives) history of cancer, menstrual and reproductive factors, and use of OC and HRT.

In order to assess the usual diet during the 2 years preceding cancer diagnosis (for cases) or hospitalization (for controls), a valid and reproducible food frequency questionnaire (FFQ) [43,44,45] was used. Subjects were asked to specify their average weekly consumption of 78 food items or food groups. Open questions allowed to collect data on other foods/recipes consumed at least once a week. A few questions aiming at assessing the patterns of dietary fat consumption were included in the FFQ. Intakes lower than once a week, but at least once per month, were coded as 0.5/week. Nutrient and total energy intake were determined according to an Italian food composition database [46]. For GI, we mainly used international nutritional tables [47]; Italian sources were used for a few local recipes [48]. We calculated the average daily GL by summing up the products of the available carbohydrate content per serving for each food or recipe, times the mean number of servings of that food per day, times the food’s GI. Therefore, each GL unit represents the equivalent of 1 g of carbohydrate from white bread [49]. We calculated the DRRD score according to Kang et al. [39], except for the replacement of GI with GL and the exclusion of trans fats that were not included in the Italian food composition tables. We used GL instead of GI because, according to the World Cancer Research Fund and the American Institute for Cancer Research, there is a “probable” association between GL (not GI) and endometrial cancer risk [50]. Thus, the DRRD score was based on the following eight dietary components: cereal fiber, coffee (caffeinated and decaffeinated), total fruit, nuts, ratio of polyunsaturated to saturated fats, GL, red and processed meat, and sweetened beverages and fruit juices. We assigned scores between 1 and 5 according to quintiles of consumption (derived from controls), in ascending order for components associated with low diabetes risk (cereal fiber, coffee, total fruit, and polyunsaturated:saturated fats ratio), and in descending order for components associated with high diabetes risk (GL and red/processed meat). The consumption of sugar-sweetened beverages and fruit juices was relatively infrequent in our population (57.0% did not consume them); therefore, we assigned a score of 5 to non-drinkers, a score of 3 to drinkers of ≤3 drinks per week (i.e., the median value among drinking controls), and a score of 1 to drinkers of more than 3 drinks per week. The consumption of nuts was reported in an open-end question of the FFQ; women declaring nuts consumption (n=15) were given a score of 2; otherwise, a score of 1 was assigned. For each woman, the overall DRRD score was calculated by summing up the scores obtained in all the dietary components. The theoretical score range was from 8 to 37, with higher values indicating greater adherence to the DRRD.

Data analysis. We used logistic regression models to calculate the odds ratios (OR) of endometrial cancer and the corresponding 95% confidence intervals (CI) for high (DRRD score >24 points, i.e., the approximate third tertile among controls) versus medium–low adherence to the DRRD, as well as for one SD increment in the score. Models were conditioned on center and quinquennia of age and adjusted for years of education (<7, 7–11, ≥12), year of interview (<1999, 1999–2003, >2003), BMI (<25, 25–29.9, ≥30 kg/m^2^), occupational physical activity (heavy/very heavy, moderate, standing or mainly sitting), smoking status (never, former, current smoker), alcohol intake (never drinker, ≤7, >7 drinks/week), history of diabetes (yes, no), total energy intake (quintiles derived from controls), age at menarche (<11, 11–13, 14–16, ≥17 years), parity (0, 1, ≥1 child(ren)), menopausal status (yes, no), use of OC (yes, no), and use of HRT (yes, no). A few missing data on adjustment factors were replaced by the median value (continuous variables) or mode category (categorical variables) according to case/control status.

In sensitivity analyses, we excluded women with diabetes and we included further adjustment for total vegetable intake. Subgroup analyses by menopausal status, parity, BMI, and smoking status were performed. We assessed heterogeneity across strata using likelihood ratio tests by comparing models with and without interaction terms for the score variable and the subgroup factors.

In addition, we calculated the OR of endometrial cancer for the combination of categories of the DRRD score and tertiles of vegetable intake. The combination of adherence to the DRRD and tertiles of vegetable intake identified six distinct categories. The category associated with the highest endometrial cancer risk (i.e., medium–low adherence to the DRRD and first tertile of total vegetable intake) was used as the reference category.

All the analyses were performed with SAS software version 9.4 (SAS Institute, Inc., Cary, NC, USA).

## 3. Results

The characteristics of endometrial cancer cases and matched controls are shown in Table 1. By design, cases and controls were of similar age and came from the same centers. Cases had a higher BMI and more commonly reported a history of diabetes. No significant differences were observed in the other analyzed factors. In our data, the DRRD score ranged from 11 to 32.

**Figure 1:**
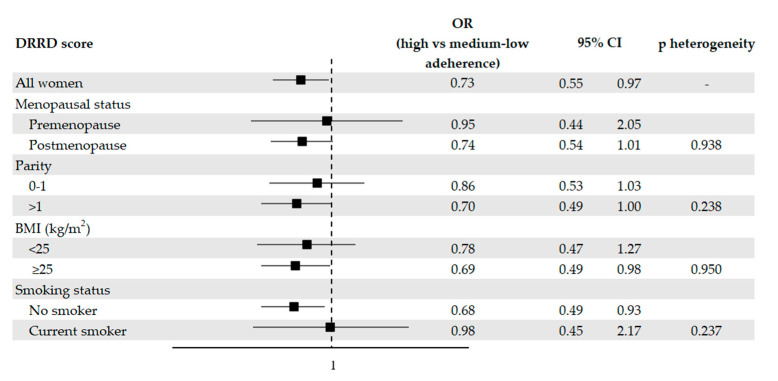
Odds ratios (OR, ■) of endometrial cancer for high versus medium–low adherence to the diabetes risk reduction diet (DRRD), with corresponding 95% confidence intervals (CIs), in the overall population and in selected subgroups, Italy, 1992–2006. OR were derived from logistic regression models, conditioned on center and quinquennia of age, and adjusted for years of education, year of interview, body mass index (BMI), occupational physical activity, smoking status, alcohol intake, history of diabetes, total energy intake, age at menarche, parity, menopausal status, use of oral contraceptives, and use of hormone replacement therapy, unless the variable was the stratification factor. P values for heterogeneity were obtained from likelihood ratio tests.

Figure 1 provides the OR of endometrial cancer with the corresponding 95% CI according to the DRRD score, in the overall population and in strata of relevant factors. 119 cases (26.2%) and 295 controls (32.5%) were highly adherent to the DRRD. Adherence to the DRRD was associated with a reduction in the risk of endometrial cancer. After adjustment for possible confounders, the OR were 0.73 (95% CI, 0.55–0.97) for high versus medium–low adherence and 0.69 (95% CI: 0.41–1.17) for one SD increment in the DRRD score. When we excluded diabetic women, we obtained an OR for high versus medium–low adherence to the DRRD of 0.75 (95% CI, 0.56–1.00). A suggestive reduced risk of endometrial cancer of borderline statistical significance was observed after adjusting for total vegetable consumption, with an OR of 0.80 (95% CI, 0.60–1.07).

Inverse associations were observed in most of the subgroups analyzed (Figure 1), with the exception of pre-menopause and current smoking subgroups, where the OR approached unity. However, there was no significant heterogeneity across strata as tested by the likelihood ratio tests. 

**Figure 2:**
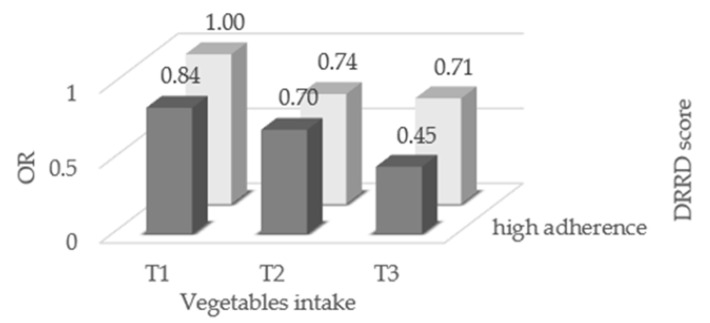
Odds ratios (OR) of endometrial cancer for combination of adherence to the diabetes risk reduction diet (DRRD) and consumption of total vegetables, Italy, 1992–2006. OR were derived from logistic regression models, conditioned on center and quinquennia of age, and adjusted for years of education, year of interview, body mass index, occupational physical activity, smoking status, alcohol intake, history of diabetes, total energy intake, age at menarche, parity, menopausal status, use of oral contraceptives, and use of hormone replacement therapy.

Single components of the DRRD score were not significantly associated with endometrial cancer risk, with the exception of a direct association with cereal fiber intake (Supplementary Materials, Table S1).The OR of endometrial cancer for the combination of adherence to the DRRD and vegetable consumption are given in Figure 2. High adherence to the DRRD combined with high consumption of vegetables significantly reduced the risk of endometrial cancer (OR 0.45, 95% CI, 0.28–0.73).

## 4. Discussion

In the current study, a score measuring adherence to a diet developed for diabetes risk reduction was inversely associated with endometrial cancer risk. After adjusting for several possible confounders, including factors related to endogenous estrogen exposure, BMI, and total energy intake, women with a high DRRD adherence score had a 27% reduced risk of endometrial cancer, in comparison to those with a medium–low adherence. The association was attenuated after allowance for vegetable intake (non-significant 20% reduced risk) and was not detected among pre-menopausal women and women who smoked. The null results in those subgroups have to be interpreted with caution given the limited number of pre-menopausal women and current smokers in our study, and may be, in any case, chance findings. Combining high adherence to the DRRD and high vegetable intake was associated with a greater decrease in the risk of endometrial cancer.

This is the first study in which the DRRD was examined in association with endometrial cancer. An inverse association of the DRRD with breast cancer [39,40], pancreatic cancer [41], and hepatocellular carcinoma [38] has previously been observed.

Epidemiological evidence on the impact of dietary factors on endometrial cancer risk is largely inconclusive [50]. With reference to dietary factors included in the DRRD, coffee was inversely related to risk of endometrial cancer [20,21]. Caffeine increases sex-hormone-binding globulin levels, and, accordingly, the concentrations of sex steroids and, therefore, endometrial hyperproliferation is reduced [51]. More relevantly, coffee could be considered an insulin sensitizer, especially among overweight and obese women [52]. Among the other favorable components of the DRRD, while some studies suggested a favorable role of high fruit consumption [36], other studies did not find any appreciable association [30,32,53]. In two meta-analyses published in 2018 and 2020 [34,54], there was an inverse association between the intake of total fibers with the risk of endometrial cancer, but it was restricted to case–control studies. In addition, in the meta-analysis published in 2018, there was a direct association with the intake of cereal fibers, based on three cohort studies [34]. Dietary fats may affect estrogen levels and obesity [55] although investigations on dietary fats provided inconsistent results. No association [56,57,58] or a weak inverse association [59] with monounsaturated fat intake was observed in some case–control studies. An association with total, saturated, and animal fats emerged in case–control studies but not in cohort studies [56]. The role of dietary GI and GL on endometrial cancer risk is still uncertain. Some studies showed a direct association of high GL diets [23] or high GI diets [60,61] with endometrial cancer risk, whereas others suggested no association [25,26,27,62,63]. While some studies observed that high red and processed meat intakes increased the risk of endometrial cancer [28,29,32], others found that the direct association was restricted to red meat only [30], and one [31] even reported inverse associations. Therefore, evidence on this topic is still controversial. The few studies investigating nut [32,64,65,66] and sugar-sweetened beverage consumption [32,67,68] in relation to endometrial cancer gave inconsistent results.

High vegetable consumption was consistently reported as protective against endometrial cancer risk in case–control studies [30,36,53]. The favorable role of vegetables may be attributable to their contents of dietary fiber, vitamins, minerals, and other micro-components such as polyphenols (flavonoids, lignans, and phenolics), phytosterols, isothiocyanates (e.g., sulforaphane from brassica vegetables), and indoles, which may have anti-inflammatory, antioxidant, and anticarcinogenic properties and may influence the modulation of steroid hormone concentrations and metabolism. Nevertheless, the inverse association was not confirmed in prospective cohort studies [69,70].

In the assessment of the association between diet and chronic diseases, the investigation of dietary patterns represents a complementary approach to the analysis of the role of distinct foods or nutrients. The dietary pattern approach allows to take into account the biologic interactions among nutrients. Dietary patterns are particularly useful when the standard method of focusing on individual foods or nutrients does not reveal significant associations [71]. Along this line, we found an inverse association with the overall DRRD, in the absence, however, of consistent associations with the single components of the score. Dietary patterns that may mediate estrogen levels and reduce chronic inflammation and that are characterized by a combination of foods rich in fibers, antioxidants, unsaturated fatty acids, and phytochemicals, such as the Mediterranean diet, have been suggested to play a beneficial role on endometrial cancer [72,73]. Conversely, in a postmenopausal women cohort study, a better diet quality, as assessed by four different a priori diet quality indices, did not influence endometrial cancer risk [74]. Again, in a case–control study conducted in the USA, different dietary patterns, including the Mediterranean diet, were not associated with endometrial cancer risk. As for the Mediterranean diet, the null results may be due to the low adherence to the Mediterranean dietary pattern in that population [75]. The DRRD and the Mediterranean dietary patterns share some common features, such as the high consumption of fruit and the low consumption of meat (both red and processed); in our data, the correlation coefficient between the two dietary patterns was 0.30.

The relationship between diabetes and endometrial cancer risk has been modulated by insulin and insulin growth factor (IGF) levels, which are likely affected by the DRRD. However, insulin and other anti-diabetic drugs have not been consistently associated with the risk of endometrial cancer [76].

There are strengths and weaknesses of our study. Generally, case–control studies are more prone to selection and information bias when compared to cohort studies. However, the almost complete participation of elected women and the exclusion from the control group of women admitted for hormone-related or gynecological conditions, or any medical condition leading to long-term dietary changes weigh against a major role of selection bias in the present study. Moreover, the recruitment areas were similar for cases and controls. Further, in the carcinogenesis of endometrial cancer, estrogens, obesity, and the metabolic syndrome seem to act on the later stages of the carcinogenic process. Along this line, a reduction in the risk of endometrial cancer was observed shortly after bariatric-surgery-induced weight loss [77]. Case–control studies collect information about exposures that occurred in the period before diagnosis and may better evaluate diet closer to cancer incidence than cohort studies, particularly those with a single exposure measurement in the distant past. Among limitations, the content of *trans* fats in Italian foods is not available from food composition tables, therefore we could not include it in the DRRD score [39]. The major strengths of our investigation were the large dataset, the use of an FFQ that showed good results when tested for reproducibility and validity [43,44,45], and the fact that we were able to adjust for several potential confounding factors.

## 5. Conclusions

In conclusion, this study suggests that high adherence to a diabetes prevention diet is inversely related to endometrial cancer risk. Combining such a diet with high vegetable intake may provide additional benefit in endometrial cancer prevention.

## Figures and Tables

**Table 1:** Distribution of cases with endometrial cancer cases and matched controls according to selected covariates. Italy, 1992–2006.

	Cases (*n* = 454) *n* (%)	Controls (*n* = 908) *n* (%)	χ^2^ (*p*-Value)
Center			
Milan	140 (30.8)	280 (30.8)	Matching variable
Naples	77 (17.0)	154 (17.0)	
Pordenone	237 (52.2)	474 (52.2)	
Age (years)			
<50	67 (14.8)	134 (14.8)	Matching variable
50–54	59 (13.0)	118 (13.0)	
55–59	81 (17.8)	162 (17.8)	
60–64	84 (18.5)	167 (18.4)	
65–69	82 (18.1)	165 (18.2)	
≥70	81 (17.8)	162 (17.8)	
Education (years)			
<7	263 (57.9)	553 (60.9)	
7–11	119 (26.2)	225 (24.8)	
≥12	72 (15.9)	130 (14.3)	1.17 (0.56)
BMI (kg/m^2^) ^a^			
<25	174 (38.3)	504 (55.5)	
25–29.9	112 (24.7)	260 (28.8)	
≥30	168 (37.0)	140 (15.5)	86.2 (<0.001)
History of diabetes			
No	401 (88.3)	854 (94.1)	
Yes	53 (11.7)	54 (6.0)	13.71 (<0.001)
Parity (number of children)			
0	68 (15.0)	126 (13.9)	
1	92 (20.3)	150 (16.5)	
≥2	294 (64.7)	632 (69.6)	3.69 (0.16)
Menopausal status ^a^			
No	85 (19.2)	174 (19.3)	
Yes	358 (80.8)	726 (80.7)	0.004 (0.95)
Smoking status ^a^			
Never smoker	331 (72.9)	646 (71.2)	
Ex-smoker	48 (10.6)	104 (11.5)	
Current smoker	75 (16.5)	155 (17.1)	0.39 (0.82)

^a^ The sum does not equal the total because of missing data.

## Data Availability

The dataset used and analyzed during the current study is available from the corresponding author on reasonable request.

## Supplementary Materials

The following are available online at https://www.mdpi.com/article/10.3390/nu13082630/s1, Table S1. Odds ratios (OR) of endometrial cancer, with corresponding 95% confidence intervals (CI), according to the single components of the diabetes risk reduction diet (DRRD). Italy, 1992–2006.
