# Supplementary material for: Diabetes Risk Reduction Diet and Endometrial Cancer Risk
Source: Nutrients. 2021 Jul 30;13(8):2630. doi: 10.3390/nu13082630 (PMC8399314; doi:10.3390/nu13082630)
Supplement: Supplementary file 1 [file nutrients-13-02630-s001.zip › nutrients-1284040-SI.pdf]

Table S1. Odds ratios [a] (OR) of endometrial cancer, with corresponding 95% confidence intervals (CI), according to the single components of the diabetes risk reduction diet (DRRD). Italy, 1992-2006.

	Quintiles of intake[b]				
	1[c]	2	3	4	5
**Cereal fiber**					
OR	1.00	1.43	1.59	1.44	1.72
(95% CI)		(0.93-2.20)	(1.01-2.52)	(0.89-2.32)	(1.04-2.84)
**Fruit**					
OR	1.00	0.57	0.87	0.96	0.75
(95% CI)		(0.38-0.85)	(0.59-1.27)	(0.65-1.41)	(0.50-1.14)
**Coffee**					
OR	1.00	1.13	1.11	1.11	0.79
(95% CI)		(0.76-1.67)	(0.76-1.63)	(0.74-1.65)	(0.52-1.20)
**PS ratio**					
OR	1.00	1.17	1.05	0.87	0.88
(95% CI)		(0.80-1.72)	(0.71-1.55)	(0.58-1.30)	(0.58-1.34)
**Glicemic load**					
OR	1.00	0.77	0.87	0.77	0.84
(95% CI)		(0.49-1.19)	(0.53-1.41)	(0.45-1.30)	(0.45-1.56)
**Red and processed meat**					
OR	1.00	1.06	1.33	1.67	1.04
(95% CI)		(0.69-1.62)	(0.87-2.03)	(1.10-2.55)	(0.65-1.66)
	Non-consumers	≤Median[b,d]	>Median		
**Soft drink and juice**					
OR	1.00	1.15	0.93		
(95% CI)		(0.85-1.55)	(0.66-1.31)		
	Non-consumers	Consumers			
**Nuts**					
OR	1.00	0.71			
(95% CI)		(0.21-2.41)			

PS: polyunsaturated:saturated fatty acids.

[a] Estimated from conditional logistic regression, conditioned on quinquennia of age and center, and adjusted for year of interview, education, total energy intake, body mass index, occupational physical activity, smoking status, alcohol intake, history of diabetes, age at menarche, parity, oral contraceptive use, hormone replacement therapy use, and menopausal status. [b] Derived among controls. [c] Reference category. [d] Median value: 3 drinks per week.
